# Heterogeneous Cerebral Vasoreactivity Dynamics in Patients with Carotid Stenosis

**DOI:** 10.1371/journal.pone.0076072

**Published:** 2013-09-27

**Authors:** Ting-Yu Chang, Wan-Chun Kuan, Kuo-Lun Huang, Chien-Hung Chang, Yeu-Jhy Chang, Ho-Fai Wong, Tsong-Hai Lee, Ho-Ling Liu

**Affiliations:** 1 Department of Neurology, Stroke Center, Chang Gung Memorial Hospital, Linkou Medical Center and College of Medicine, Chang Gung University, Taoyuan, Taiwan; 2 Department of Medical Imaging and Radiological Sciences, Chang Gung University, Taoyuan, Taiwan; 3 Department of Medical Imaging and Intervention, Chang Gung Memorial Hospital, Taoyuan, Taiwan; 4 Healthy Aging Research Center, Chang Gung University, Taoyuan, Taiwan; University of Maryland, College Park, United States of America

## Abstract

Cerebral vasoreactivity (CVR) can be assessed by functional MRI (fMRI) using hypercapnia challenges. In normal subjects, studies have shown temporal variability of CVR blood oxygenation level-dependent responses among different brain regions. In the current study, we analyzed the variability of BOLD CVR dynamics by fMRI with a breath-holding task in 17 subjects with unilateral carotid stenosis before they received carotid stenting. Great heterogeneity of CVR dynamics was observed when comparing BOLD responses between ipsilateral and contralateral hemispheres in each patient, especially in middle cerebral artery (MCA) territories. While some subjects (n=12) had similar CVR responses between either hemisphere, the others (n=5) had a poorly correlated pattern of BOLD changes between ipsilateral and contralateral hemispheres. In the latter group, defined as impaired CVR, post-stenting perfusion tended to be more significantly increased. Our data provides the first observation of divergent temporal BOLD responses during breath holding in patients with carotid stenosis. The development of collateral circulation and the derangement of cerebral hemodynamics can be detected through this novel analysis of the different patterns of BOLD changes. The results also help in prediction of robust increase of perfusion or hyperperfusion after carotid stenting.

## Introduction

Cerebral vasoreactivity (CVR), mostly assessed when cerebral perfusion increases after vasodilatory stimuli, has been recognized as a sensitive indicator for the integrity of cerebral hemodynamic responses. Previous researches have documented that the impairment of CVR might be related to various conditions including hypertension [1], cognitive impairment [2,3], diabetes mellitus [4], and even sleep apnea syndrome [5]. Carotid stenotic or occlusive disease is considered to have the greatest influence on CVR [6], which represents the reserve capacity of cerebral perfusion and often begins deteriorating before a clinical ischemic event under compromised hemodynamics. It is suggested that evaluating CVR in patients with carotid stenosis may help to identify the higher risk of stroke in these patients and a better timing of interventional treatment [7,8].

In the past few decades, measurement of increased CBF by single photon emission computed tomography (SPECT) analysis after administration of vasodilating agents had been the most popular method to interpret CVR [9,10]. The advantage of SPECT is that it can be used to quantitatively calculate blood flow (since we know that CVR represents the amount of perfusion change per each mmHg of end-tital carbon dioxide (CO2) elevation). However, this method is relatively time consuming, with compromised spatial resolution, and involves radiation load. Recent studies have effectively employed arterial spin labeling (ASL) MRI as a totally non-invasive method for quantitative assessment of CVR in patients with carotid artery stenosis/occlusion [11,12]. In addition to ASL, functional magnetic resonance imaging (fMRI) based on blood oxygenation level-dependent [13] can serve as an alternative method for assessing CVR. Elevated arterial CO2 concentration by CO2 inhalation or breath-holding task leads to vasodilatory responses and is followed by increased global cerebral perfusion. Then, the paramagnetic deoxyhemoglobin concentration reduces, which leads to a rise in BOLD signal in T2*-weighted MR images [14]. Though fMRI is safe, non-invasive, time-saving, and with good spatial resolution, it should be used with caution for evaluating CVR: first, latencies of peak BOLD responses can vary between different vascular territories or brain regions [15,16], and this may cause inter-subject variation; second, as in previous studies [17,18], using normalized BOLD changes as simple ratios to represent CVR is not quantitative and seems an oversimplification when applied in complex conditions such as carotid stenosis, in which the development of rich collateral circulation would modify hemodynamics; and third, the BOLD signal intensity varies following the alteration of end-tidal partial pressure of CO2 (EtPCO_2_), which may change not only among subjects but also with time in the same person [19].

Temporal variability of the BOLD responses derived from CVR of different brain regions can have great implications on fMRI for causality and connectivity analyses. Leoni et al. showed significantly varied CVR responses from different vascular territories [16]. Bright et al. proposed a cued deep breathing task for studying the magnitude and timing of the BOLD CVR response and found significant regional heterogeneity of the CVR dynamics in the brain [20]. More recently, Blockley et al. used Fourier analysis techniques to characterize the delays of the BOLD CVR response, and showed that the frontal and parietal lobes reacted earlier than the occipital lobe [21]. Murphy et al. emphasized the importance of measuring EtPCO_2_ when performing analysis of CVR based on different brain regions [22]. In patients with carotid stenosis, a previous study found delayed BOLD response with decreased amplitude in the motor cortex as evoked by a motor task paradigm [23]. To the best of our knowledge, no published studies have examined CVR dynamics with a hypercapnia paradigm in diseased subjects. The aim of the current study was to investigate the variability of the BOLD CVR dynamics in patients with significant unilateral carotid stenosis undergoing carotid angioplasty with stenting (CAS). This study also evaluated the potential utility of the CVR heterogeneity as an indicator for predicting the changes in cerebral perfusion after CAS.

## Materials and Methods

### Subjects

Seventeen patients with unilateral internal carotid artery (ICA) stenosis (≥60% according to the North American Symptomatic Carotid Endarterectomy Trial NASCET criteria) who received CAS were enrolled for this study (Table 1). All subjects were males, and the mean age was 74.2±6.56 (56–83) years old. Exclusion criteria included interventional coronary or peripheral artery treatment in the past 30 days, stroke within the past 3 weeks, a previous stroke producing (≥one-third) middle cerebral artery (MCA) territory infarction, significant (≥50%) intracranial stenosis or contralateral ICA stenosis, allergy to iodinated or gadolinium-based contrast medium, and inability to perform a breath-holding task cooperatively. All subjects had a series of tests including brain CT, color-coded carotid duplex and transcranial Doppler sonography, and digital subtraction angiography (DSA) before the inverventional treatment. Blood pressure was carefully monitored during the periprocedural period, and the averaged mean arterial pressure (MAP) on the day before intervention was 93.7±8.5 mmHg.

**Table 1:** Demographic data of the subjects.

No.	Age	HTN	Smoking	DM	CAD	Side	Stenosis*	BIF	MRS	MAP**
1	73	Y	Y	N	Y	L	70	N	0	89.7
2	78	Y	Y	N	Y	R	68	Y	1	79.2
3	81	Y	Y	N	Y	R	81	N	1	106.5
4	70	Y	Y	N	Y	L	60	N	1	103.5
5	70	Y	Y	N	Y	R	65	Y	0	106.2
6	77	Y	N	N	Y	R	80	Y	1	93.9
7	67	Y	N	Y	Y	L	70	N	1	108.1
8	56	N	N	N	N	R	89	N	0	81.9
9	75	Y	Y	N	N	R	91	N	2	89.3
10	82	Y	N	N	Y	L	89	N	0	95.3
11	79	Y	N	N	N	R	67	N	1	95.6
12	76	Y	Y	Y	N	R	70	N	0	83.7
13	73	Y	Y	N	Y	R	90	N	1	89.8
14	83	Y	Y	N	Y	R	92	Y	1	91.8
15	78	Y	Y	N	Y	R	81	N	0	91.7
16	70	Y	Y	N	Y	L	80	Y	1	89.7
17	73	Y	Y	Y	Y	R	91	Y	1	97.1
Mean	74.2	17/17	12/17	3/17	13/17		78.5	6/17	0.7	93.7

* stenosis (%) ; ** MAP (mmHg); HTN: hypertension; DM: diabetes mellitus; CAD: coronary artery disease; BIF: plaque with carotid bulb involved; MRS: modified Rankin scale; MAP: mean arterial pressure

### Ethics Statement

Written informed consents were obtained from all patients. This study was conducted according to the principles expressed in the Declaration of Helsinki and was approved by the Institutional Review Board of Chang Gung Memorial Hospital.

### MRI acquisition

Before intervention, BOLD MRI was conducted on patients for dynamic CVR evaluation using a breath-holding paradigm. Dynamic susceptibility contrast- (DSC-

) MRI was performed both prior to and 3-5 days after CAS for evaluation of the cerebral perfusion.

MRI studies were conducted on a 1.5 Tesla scanner (Gyroscan Intera, Philips, Best, the Netherlands). The scanning protocols included anatomical sequences, DSC perfusion imaging, and BOLD MRI. The anatomical images included axial T1-weighted images (spin-echo, TR/TE=449/12 ms), axial T2-weighted images (fast spin-echo, TR/TE=4000/90 ms, ETL=17), and fluid-attenuated-inversion recovery (FLAIR: TR/TE/TI=9416/90/2200 ms). Twenty axial slices (slice thickness=5 mm and inter-slice gap=1.5 mm) were acquired to cover the whole brain. All the imaging studies were performed with the same dimensions, i.e. number of slices, slice thickness, and locations. For the dynamic BOLD study, the breath-holding paradigm was applied according to the conditions specified in our previous studies [24]. Briefly: 1) patients were made to breathe naturally for at least 6 minutes before breath-holding task; 2) during fMRI scanning, patients were asked to hold their breaths for 15 seconds after last expiration and then to breathe naturally for the next 45 seconds. This cycle was repeated for 4 times. There was no hyperventilation period. A respiratory belt was fastened across each patient’s chest to ensure that the patients followed the breathing instructions properly. BOLD MRI was performed using a T2-weighted single-shot gradient echo EPI sequence with the following parameters: TR/TE/FA=3000 ms/50 ms/90°, matrix size=112×84 and FOV=192 mm×192 mm. During the breath-holding procedure, 80 dynamic measurements were obtained with a total scan time of 4 minutes. For DSC-MRI, a single-shot gradient echo EPI sequence was applied with the following parameters: TR/TE/FA=1500 ms/40 ms/55°, matrix size=112×84 and FOV=192 mm×192 mm. Sixteen milliliters of gadolinium-diethylene-triamine penta-acetic acid (Gd-DTPA: Magnevist, Schering, Berlin, Germany) was injected into patients using a power injector with an injection rate of 4 mL/s.

### Image Interpretation and Analysis

#### Determination of region of interest (ROI)

**Figure 1:**
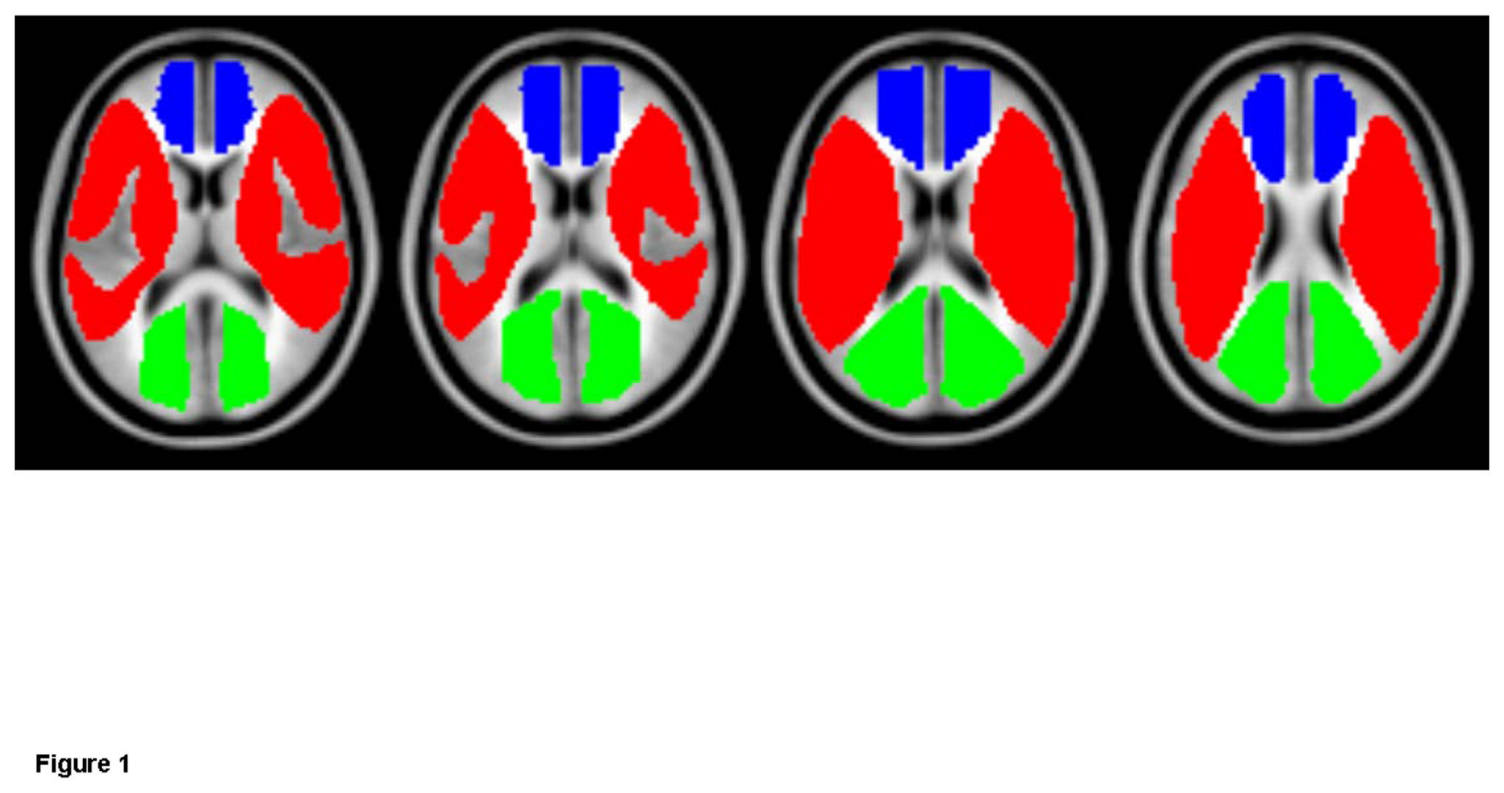
Colored map of the region-of-interest template (selected sections from basal ganglia to centrum semiovale, left to right, in transverse view). Blue and red represent the ACA and MCA territories, respectively. Occipital white matter, which is shown in green, is used as a reference region.

The T1-weighted images of each subject were normalized to the standard Montreal Neurological Institute (MNI) brain template using the statistical parametric mapping 2 (SPM2) software (Wellcome Department of Imaging Neuroscience) [25]. The normalized images were then averaged from the data for all the patients in order to determine the ROI template. The ROIs were manually placed in bilaterally symmetric regions of the anterior cerebral artery (ACA), middle cerebral artery (MCA) and posterior cerebral artery (PCA) territories by an experienced neurologist. In addition, ROIs were also placed in the occipital white matter (OWM) for reference (Figure 1).

#### Data analysis of CVR

All BOLD images were motion corrected, spatially normalized to the MNI template and spatially smoothed with a Gaussian kernel (FWHM=5 mm) using SPM2 software. For each patient, averaged signal time curves in the ACA, MCA and PCA territories of the ipsilateral and the contralateral hemisphere, relative to the stenotic side, were determined using the above-mentioned ROI template to generate corresponding masks. Furthermore, the Pearson correlation coefficient between the time courses of the ipsilateral and the contralateral MCA territories were calculated [26] and transferred to the t-score. The correlation between the two time courses was considered significant if the t-score was greater than 2.1 (r^2^>0.18, p<0.05).

#### Data analysis of perfusion

Perfusion images were analyzed using Nordic Image Control and Evaluation (ICE) software (Nordic Imaging Lab, Norway). Arterial input functions were carefully chosen from the contralateral MCA to estimate relative CBF maps using the singular value decomposition (SVD) deconvolution algorithm [27]. The above-mentioned ROI template was spatially transferred to each patient’s image space, using SPM2, to determine averaged relative CBF values in relevant regions. For each DSC-MRI study, the relative CBF values obtained in either side of the MCA regions were then divided by the mean value of the ipsilateral OWM region, and the ratios represented normalized CBF (nCBF) values. The change in the nCBF of either side of the brain was evaluated with a CBF index, calculated as follows: (nCBF after CAS)/(nCBF before CAS). A CBF index value of more than 1 represented increased blood flow after CAS.

## Results

The demographic data of 17 patients are listed in Table 1. Hypertension was the most important risk factor (all patients had hypertension). Besides, our patients also had high incidence of concomitant coronary artery disease (CAD) (13 out of a total of 17, 76%). Prior to CAS, 8 patients had previous ipisilateral cerebral infarctions and 6 patients had transient ischemic attacks. The other 3 patients had severe posture-related dizziness without definite ischemic event, and the stenotic severity of these 3 subjects was>80%. All the patients who had previously suffered strokes showed good functional recovery with modified Rankin scale score≤2. All patients underwent successful CAS procedures, and none of them developed clinical hyperperfusion syndrome (i.e. severe ipsilateral headache, seizure, or intracranial hemorrhage). No infarct developed in any patient after CAS, as was demonstrated by MRI.

**Figure 2:**
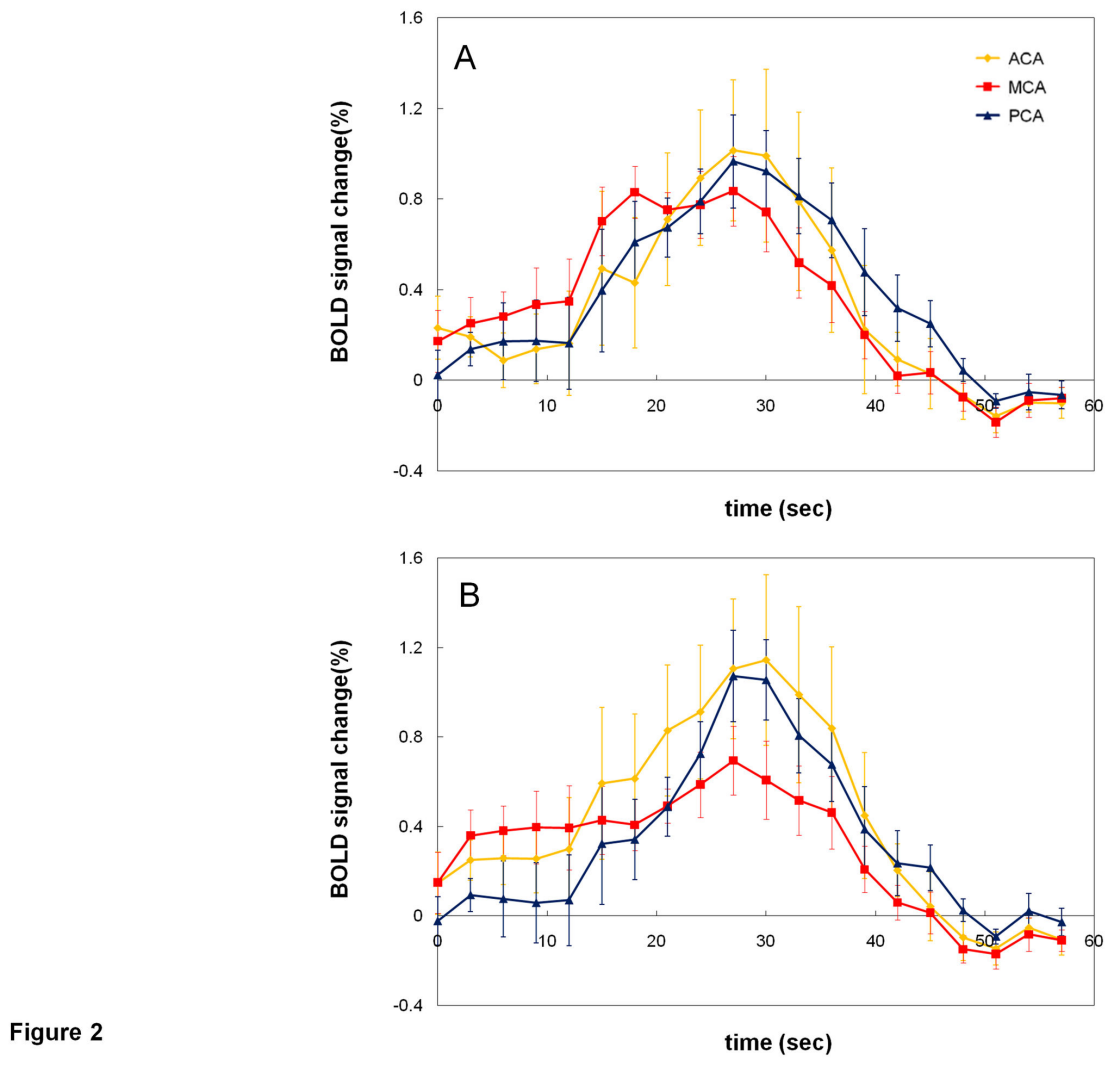
The BOLD signal time curves averaged over 17 patients from the contralateral (A) and ipsilateral (B) hemisphere.

Over seventeen patients, the curves of averaged BOLD CVR signal changes in ACA, MCA, and PCA territories were shown in Figure 2A (contralateral hemisphere) and Figure 2B (ipsilateral hemisphere). From inspection, in the contralateral side (Figure 2A), the overall BOLD response was similar to previously published studies in the normal subjects [16,28], in which MCA reached a peak the earliest, though in our study the ACA territory showed compatible response with the PCA territory. While in the ipsilateral side (Figure 2B), the BOLD CVR signal response of MCA territory showed delayed onset and smaller peak amplitude as compared to the contralateral hemisphere.

**Figure 3:**
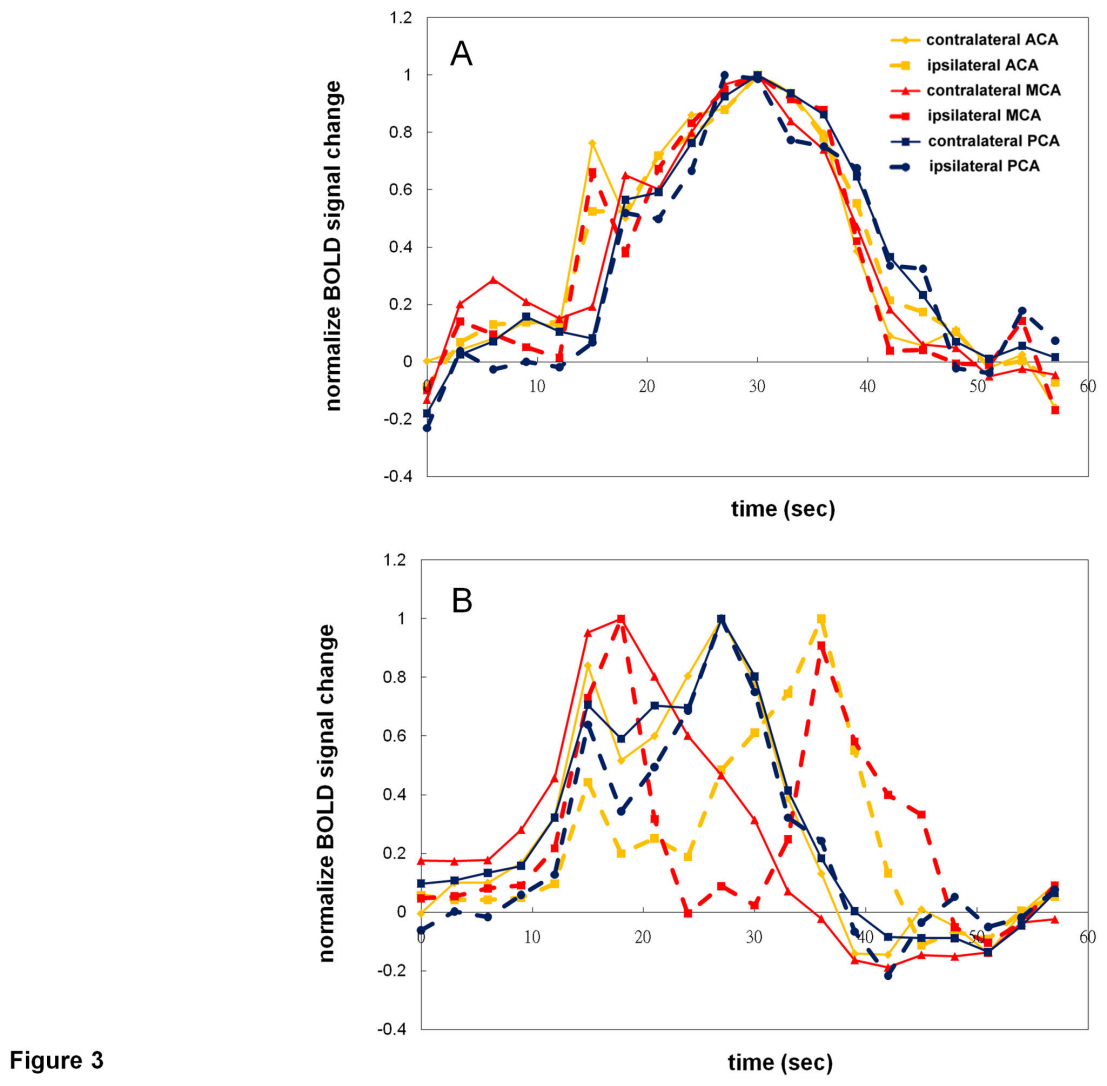
The time curves from two patients with right ICA stenosis. The BOLD responses between different ROIs, from the same breath holding task, were consistent in patient A (A), but with large varieties in patient B (B).

**Figure 4:**
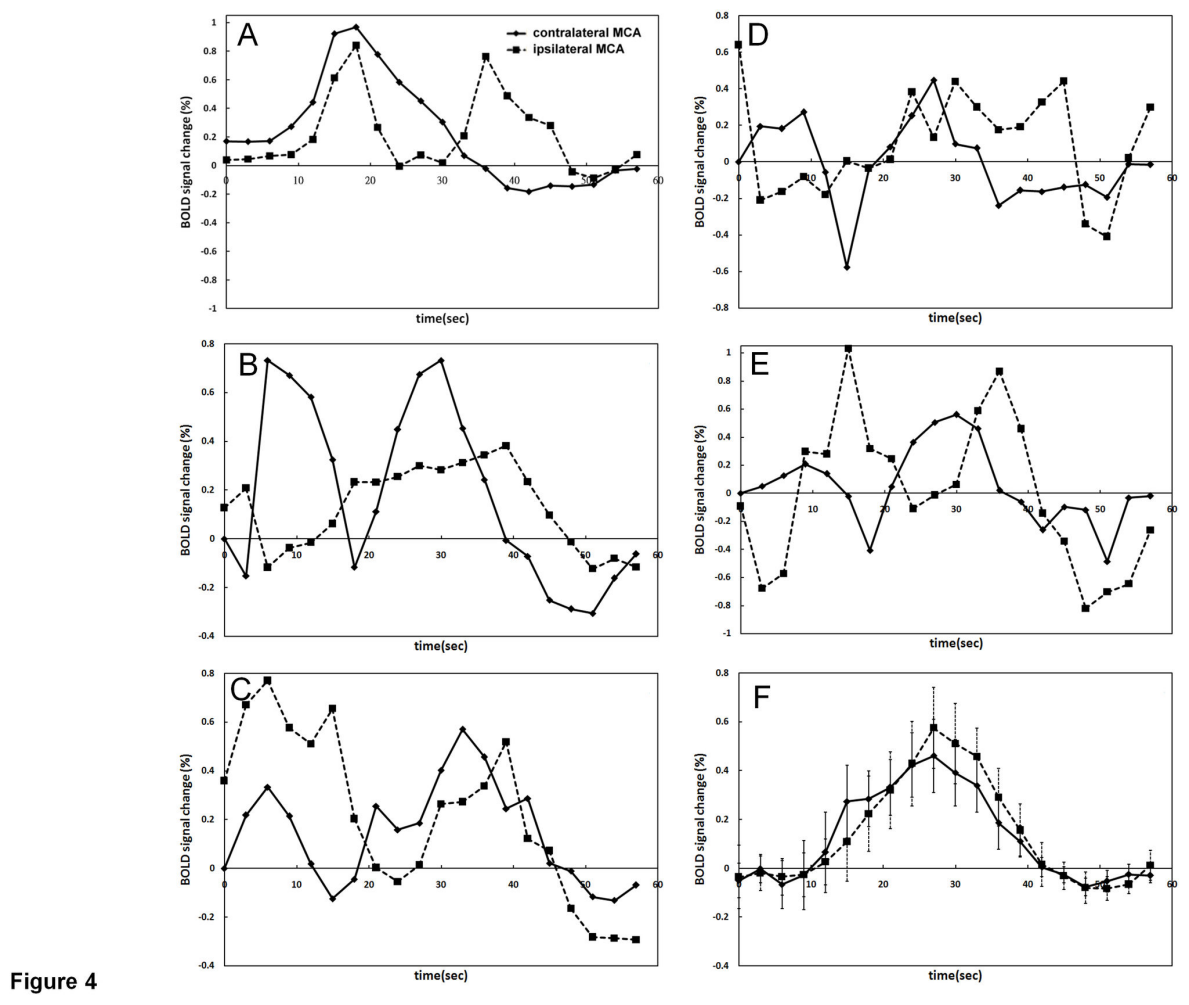
The time curves of contralateral and ipsilateral MCA BOLD signal changes from patients with MCA t-score<2.1, p>0.05 (ipsilateral side CVR poorly correlated with contralateral side CVR) (A-E). The time curves averaged from the remaining 12 patients with MCA t-score≥2.1, p<0.05 (ipsilateral side CVR well correlated with contralateral side CVR). Error bars in (F) indicate one standard deviation.

When comparing the BOLD CVR signal changes between contralateral and ipsilateral sides of different vascular territories in each subject, there was great divergence of their BOLD signal time-curve patterns. Figure 3A shows normalized BOLD CVR responses from one of the patients, which demonstrate consistent patterns in different territories and between the ipsilateral and the contralateral sides. On the other hand, responses from another patient exhibit great variations between territories and hemispheres, as illustrated in Figure 3B. In particular, consistent temporal patterns were found in the BOLD CVR responses from the ACA and PCA territories of the contralateral hemisphere, and the PCA territory of the ipsilateral hemisphere; whereas the patterns of the ACA and MCA territories of the ipsilateral hemisphere were very different in this patient. When focusing on the MCA territory, which would be most likely affected by the ICA stenosis, 12 patients had BOLD CVR responses significantly correlated between the contralateral and the ipsilateral sides (t≥2.1, p<0.05). Figure 4A-E shows each of the total five patients who had divergent MCA BOLD responses (t<2.1, p>0.05) between the two hemispheres. No consistent patterns in the differences between the two time curves could be found in these patients. This group of patients was defined as impaired CVR response of the ipsilateral hemisphere. Averaged BOLD signal time curves from the other 12 patients are demonstrated in Figure 4F, which shows relatively similar CVR responses from the two hemispheres.

In the current study, the group with inconsistent BOLD changes of ipsilateral versus contralateral MCA territories (MCA t-score<2.1) was defined as impaired CVR. When comparing clinical characteristics between patients with impaired CVR (MCA t-score<2.1, n=5) and non-impaired CVR (MCA t-score≥2.1, n=12), higher percentage of smoking (100% vs. 58.3%), co-morbidity with CAD (100% vs. 66.7%), and plaque involving carotid bulb (60% vs. 25%) was found in the former group, though these results did not reach statistical significance. Furthermore, the severity of ICA stenosis was significantly increased in patients with MCA t-score<2.1, i.e. the impaired CVR group (75.0% -*vs.*- 86.8%, *p*=0.03).

**Figure 5:**
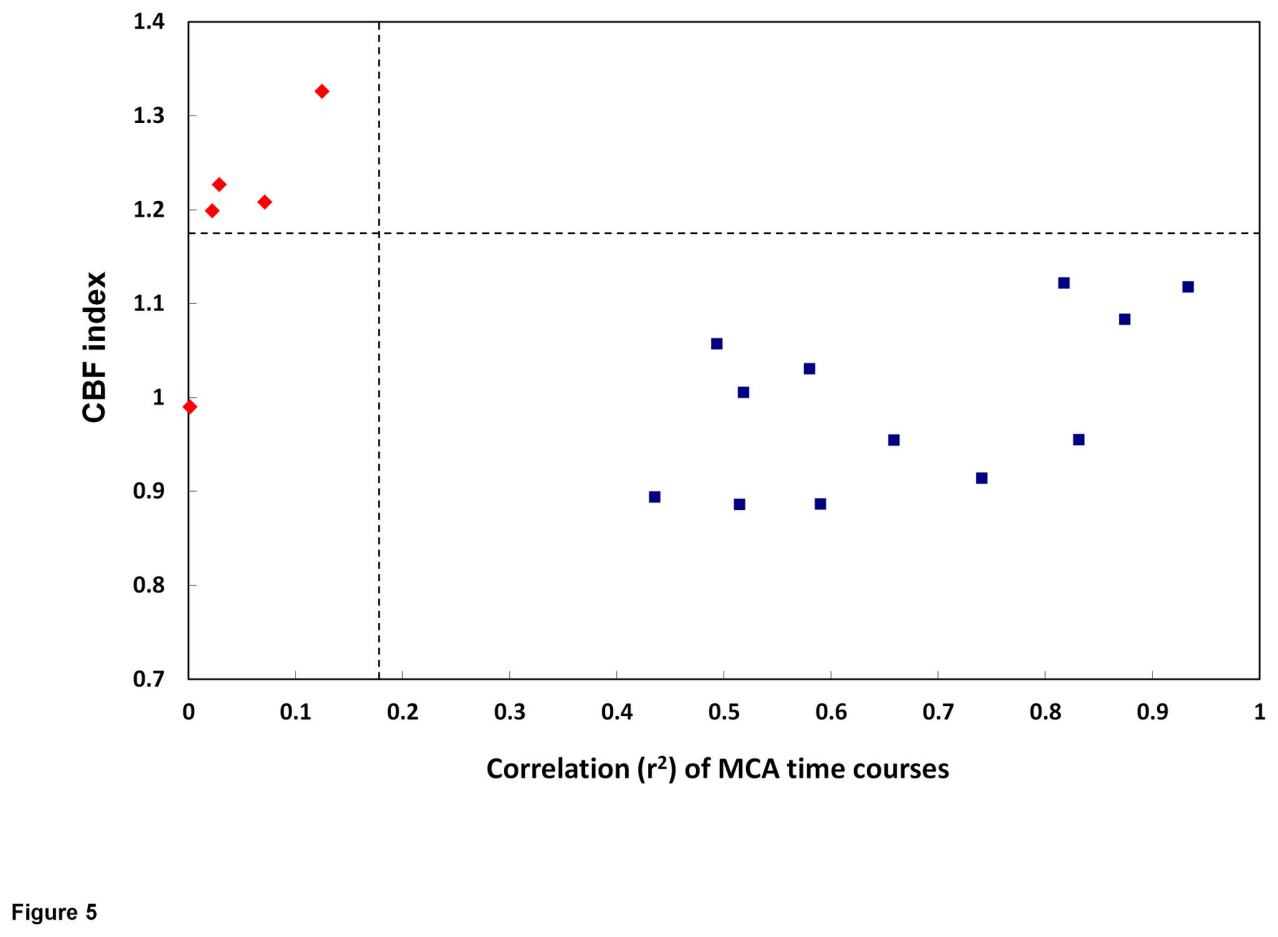
The scatter plot of CBF index of lesion side versus correlation (r^2^) of the ipsilateral and contralateral MCA time courses from the 17 patients. Red dots represent patients with r^2^<0.18 (t-value<2.1, p>0.05). Four of these five patients have greater CBF index than the patients with r^2^>0.18 (t-value≥2.1, p<0.05) by one standard deviation (CBF index>1.17).

CBF index of the ipsilateral hemisphere was applied to represent the ipsilateral CBF changes after CAS. Figure 5 shows the scatter plot of the CBF index of ipsilateral side versus the correlation (r^2^) of the ipsilateral and contralateral MCA time courses from all patients. A relationship of lack of significant correlation between MCA time courses (t<2.1) and high CBF index was found. In patient whose baseline CVR of both MCA territories was poorly correlated (i.e. t<2.1), the CBF after CAS tended to be a greater increase. As shown in Figure 5, patients could be divided into two groups according to the correlation between their MCA time courses and the cut-off level of CBF index would be 1.17, e.g. one plus one standard deviation of the group with significantly correlated MCA time courses. In our study a total of five subjects had non-significant correlation in their MCA time courses and four of them had a CBF index of more than 1.17.

## Discussion

To the best of our knowledge, this is the first study to examine dynamic hypercapnic BOLD CVR characteristics among different brain territories based on vascular supply in patients with unilateral carotid stenosis. Based on visual inspection of Figure 2A, the BOLD signal time curves of the non-stenotic side of the MCA territory showed results consistent with previous study in normal subjects [16], i.e. with the earliest onset latency, the shortest time-to-peak, and the smallest amplitude. While in the stenotic hemisphere, the curve of MCA BOLD signals among different patients showed great heterogeneity. Such variability in BOLD CVR responses may have great implications in fMRI-based causality and connectivity analyses in the patient group. This variation, mainly observed in MCA territory but not in ACA or PCA territory, may be related to the fact that blood supply of MCA is directly from ipsilateral ICA. If there is proximal stenosis of a major artery, its distal and terminal circulation would be most influenced. Though ACA is also derived from terminal ICA, hypoplasia of the A1 portion of ACA is not uncommon and there would be good collateral circulation from the anterior communicating artery[29]. Therefore, MCA territory should be more representative for evaluating hemodynamic changes in patients with significant carotid stenosis. PCA territory seems stationary in perfusion change, which means, in patients with bilateral carotid stenosis, PCA territory could be a reference area for assessing CVR

In the present study we performed analysis of the correlation between contralateral MCA and ipsilateral MCA BOLD CVR responses. A t-value<2.1 represented the CVR curve of ipsilateral MCA poorly correlated with that of contralateral MCA, and in this group CVR was presumed deteriorated. This is a novel method of estimating CVR for patients with carotid stenosis. In our previously published data [24], CVR was also evaluated based on number of voxels with significant BOLD changes over MCA territories of either hemisphere, from which a laterality index was calculated for each patient. Although that could signify the discrepancy of CVR between two hemispheres, the relative ratio of CVR might be under- or over-estimated because regions that were judged as low CVR by conventional fMRI analysis could be erroneous if the response shapes were far from the modeled response. This is because conventional fMRI analysis seeks responses having high correlations with an assumed model, but with disease (carotid stenosis) progressing and collateral circulation developing, not only the BOLD amplitude but also the temporal characteristics may change, as found in this study. There were other studies that have tried to improve the preciseness of fMRI in normal controls: some have suggested using EtCO_2_ to correct the percentage of BOLD change by per mmHg rise in EtCO_2_[22], or applying another tool such as near-infrared spectroscopy concomitantly with fMRI [30]. It should be noted that in the current study, the impairment of CVR was evaluated directly by the comparison of the dynamic pattern of BOLD change between the hemispheres. This may be more practical to implement in clinical populations since accurate estimation of the response amplitude is not required. Based on this concept, there would be no need to monitor absolute EtCO_2_ of each single patient, as long as the execution of breath-holding paradigm was warranted. This analysis is simple, easily approached for clinicians, and as mentioned above, more precisely representative for CVR in patients with carotid stenosis.

In patients with severe carotid occlusive/stenotic disease, there is a variety of clinical manifestations: some patients develop fatal ischemic strokes, while others may remain asymptomatic for a long time. The pathophysiology leading to subsequent ischemic scenario or balanced hemodynamics may be complicated, but collateral circulation has been considered a major aspect affecting the risk of stroke [31]. Brain circulation attempts to remain in constant perfusion under different physiological conditions due to the mechanism called autoregulation of blood flow [32]. When it is compromised by a proximal stenotic/occlusive artery, collateral circulations build up. However, the collateral vasculatures evaluated by advanced imaging tools often reveal inconsistent results among individuals, and the development of collateral vessels does not guarantee compatible perfusion [31]. Hence, CVR, the reserve capacity of cerebral perfusion, has been recommended as a sensitive indicator for the risk of ischemic events [33,34]. This study may not only provide a model of assessing CVR, but also a way of observing various perfusion patterns that might be related to different collateral circulations. Our subjects with impaired CVR (MCA t-score<2.1) had divergent BOLD signal patterns. As the subject shown in Figure 3B, the biphasic BOLD response of ipsilateral MCA might reflect inadequate circulation: compared to contralateral MCA, in which the BOLD responses arose, sustained and degraded gradually, at ipsilateral MCA the first BOLD peak dropped abruptly followed by the second peak. This may be associated with insufficiency of local perfusion and delayed rescue of collateral flow, which reveals compromised hemodynamics. Similar biphasic BOLD signal curves could also be observed in other subjects as seen in Figure 4A, B, C, and E. We presume that detecting impaired CVR by the current method reflects derangement of cerebral hemodynamics. This model could simultaneously evaluate deficit CVR dynamics and display vivid changes of hemodynamics, which may help to subsequently analyze the build-up of circulatory supply in different individuals.

Patients with impaired CVR showed a wide variety of BOLD pattern changes in the current study (Figure 4A-E). This divergence may be interpreted as proof that many factors play a role in CVR decline. The severity of stenosis, which was significantly higher in the impaired CVR group, might be most important, and indicated that cerebral hemodynamics deteriorate with the progression of carotid stenosis. Other than stenotic severity, a trend of higher incidence for smoking (100%), concomitant CAD (100%), and atherosclerotic plaque involving carotid bulbs (60%) in the impaired CVR group was also seen in our data. It has been documented that the impairment of endothelial dysfunction may have negative influence on CVR [35], which might explain why our patients with impaired CVR were all smokers and had concomitant CAD. The increased percentage of plaque over carotid bulbs in the impaired CVR group was another interesting finding. As we know, baroreceptors, that serve to modulate vasomotor response to regulate blood pressure and heart rate, mainly locate at the carotid bulb. This is a mechanoregulation of blood flow mediated by the autonomic nervous system. However, whether this reflex has a long-term effect on cerebral blood flow and CVR is unknown due to the paucity and inconclusiveness of data [35,36]. From our results, albeit drawn from a limited number of cases, it might be possible to speculate that baroreflex involves in some way with CVR. Further investigation with extended cases is needed.

Comparison between the correlation of temporal MCA CVR and CBF index (perfusion change) of stenotic side in each subject revealed that impaired CVR is related to high CBF index (increased perfusion). From the quadrant graph (Figure 5), there are five subjects with impaired CVR and four of them had a high CBF index (cutoff: 1.17). Our preliminary data of 17 patients showed 100% (4/4) sensitivity and 92% (12/13) specificity for predicting post-stent perfusion change by the current analysis method. This result is compatible with our previous work and other studies, i.e. the benefit of increased perfusion after intervention may be more significant in patients with poor CVR, while impaired CVR may increase the risk of hyperfusion after carotid intervention [24,37,38]. The method adopted in the current study was totally non-invasive and may be applied more easily as long as a simple breath-holding task, e.g. for 15 s, can be performed by patients.

The current study has some limitations. First, our sample size was small, and trends noted in some results need extended case numbers to observe possible statistical significance and clinical meaning. Second, the correlation analysis assumed unaffected BOLD CVR response in the normal side which could be employed only for unilateral carotid stenosis. For patients with bilateral carotid stenosis, PCA territory could be a reference area since its BOLD signal remained stationary. Third, the current study did not enroll carotid occlusive subjects. There should be some hemodynamical characteristics in this group and the pattern of its development of collateral circulation could be more distinguished and complicated.

In conclusion, this study examined dynamic BOLD CVR responses in patients with unilateral carotid stenosis by adoption of a breath-holding task. The results demonstrated significant divergence in temporal patterns of the responses between the two hemispheres in some patients. A correlation analysis was able to depict the impaired CVR and the approach was effective for predicting perfusion changes after the stenting. This study proposes that analyzing dynamic BOLD response to assess CVR heterogeneity is a potential method that could be easily and routinely applied in patients with carotid stenosis and those preparing for carotid intervention.
